# A Panel of Urinary Volatile Biomarkers for Differential Diagnosis of Prostate Cancer from Other Urological Cancers

**DOI:** 10.3390/cancers12082017

**Published:** 2020-07-23

**Authors:** Ana Rita Lima, Joana Pinto, Carina Carvalho-Maia, Carmen Jerónimo, Rui Henrique, Maria de Lourdes Bastos, Márcia Carvalho, Paula Guedes de Pinho

**Affiliations:** 1 UCIBIO/REQUIMTE, Department of Biological Sciences, Laboratory of Toxicology, Faculty of Pharmacy, University of Porto, 4050-313 Porto, Portugal; jipinto@ff.up.pt (J.P.); mlbastos@ff.up.pt (M.d.L.B.); 2 Cancer Biology & Epigenetics Group, Research Center (CI-IPOP), Porto Comprehensive Cancer Center (P.CCC), Portuguese Oncology Institute of Porto (IPO Porto), 4200-072 Porto, Portugal; carina.carvalho.maia@ipoporto.min-saude.pt (C.C.-M.); carmenjeronimo@ipoporto.min-saude.pt (C.J.); rmhenrique@icbas.up.pt (R.H.); 3 Department of Pathology, Portuguese Oncology Institute of Porto (IPO Porto), P.CCC Porto Comprehensive Cancer Center, 4200-072 Porto, Portugal; 4 Department of Pathology and Molecular Immunology, Biomedical Sciences Institute (ICBAS), University of Porto, Rua Jorge Viterbo Ferreira 228, 4050-313 Porto, Portugal; 5 Fernando Pessoa Energy, Environment and Health Research Unit (FP-ENAS), Faculty of Health Sciences, University Fernando Pessoa, 4249-004 Porto, Portugal

**Keywords:** prostate cancer, renal cancer, bladder cancer, volatile organic compounds, urinary biomarkers, detection

## Abstract

Our group recently developed a urinary 6-biomarker panel for the diagnosis of prostate cancer (PCa) which has a higher level of accuracy compared to the serum prostate specific antigen (PSA) test. Herein, urine from an independent cohort of PCa patients and cancer-free controls was analyzed to further validate the discriminative power of that panel. Additionally, urine from patients diagnosed with bladder cancer (BC) and renal cancer (RC) were included to evaluate the site-specificity of the panel. Results confirmed the ability of the 6-biomarker panel to discriminate PCa patients from controls, but not from other urological cancers. To overcome this limitation, an untargeted approach was performed to unveil discriminant metabolites among the three cancer types. A 10-biomarker panel comprising the original panel plus four new metabolites was established to discriminate PCa from controls, BC, and RC, with 76% sensitivity, 90% specificity, and 92% accuracy. This improved panel also disclosed better accuracy than serum PSA test and provides the basis for a new non-invasive early detection tool for PCa.

## 1. Introduction

Globally, prostate cancer (PCa) ranks first amongst all male urological cancers and second in incidence of all cancers in men [1]. Due to its asymptomatic nature at early stages, long latency period, and potential for cure [2], PCa is a perfect candidate for screening programs. Currently, early PCa detection is mostly based on digital rectal examination (DRE) and serum prostate specific antigen (PSA) assessment, which stratify patients for subsequent prostate biopsy [2,3]. However, these procedures have serious limitations and have led to overdiagnosis and consequent overtreatment of low-risk patients, unnecessary biopsies, and unwarranted radical prostatectomies [4]. Indeed, the standard serum PSA cut-off of 4 ng/mL has failed to meet the criteria required for an effective biomarker due to its limited sensitivity (20.5%), specificity (51–91%) [5,6], area under the curve (AUC) (0.53–0.83), and accuracy (62–75%) [7]. Based on these limitations, several research groups have proposed new candidate biomarkers for PCa detection (e.g., prostate cancer antigen 3 (PCA3) and prostate health index (PHI)) [8,9,10]. PCA3 score has 63% sensitivity, 88% specificity, and an AUC of 0.82, considering a cut-off of 35 [9]. However, the definition of the ideal cut-off for this biomarker remains controversial [8]. PHI combines total serum PSA, free PSA (fPSA), and [-2]proPSA (p2PSA) and outperforms serum PSA with an AUC ranging from 0.70 to 0.77 [10].

Metabolic rewiring has recently been recognized as a hallmark of cancer cells [11], boosting the search for innovative PCa detection strategies based on the study of tumor-associated metabolic alterations [12,13,14,15]. Volatile organic compounds (VOCs) are end products of cellular metabolism, especially promising as potential non-invasive biomarkers for translation into the clinic due to the recent advancements in the development of electronic-nose (e-nose) sensors [16]. In this vein, we have recently reported a urinary biomarker panel for PCa diagnosis comprising six volatile compounds, that outperformed PSA sensitivity and accuracy [17]. Here, we extend our previous work to further validate the biomarker panel in an independent cohort of PCa patients and also to assess the performance of the panel for discriminating PCa from other common urological cancers, namely bladder cancer (BC) and renal cancer (RC).

## 2. Results

### 2.1. Evaluation of the Diagnostic Performance of 6-Volatile Biomarker Panel

**Figure 1:**
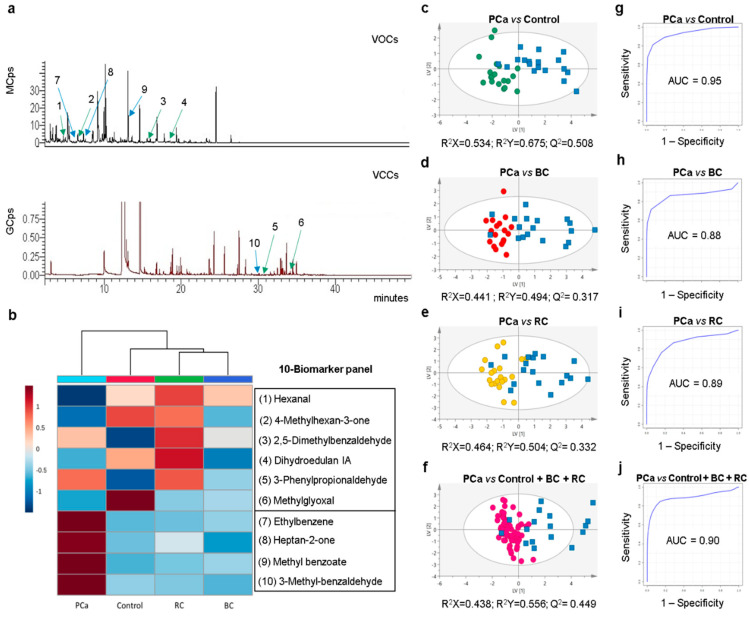
(**a**) Representative GC-MS chromatograms of volatile organic compounds (VOCs) and volatile carbonyl compounds (VCCs) present in urine of prostate cancer (PCa) patients (green arrows indicate the six volatiles (numbers 1–6) in former biomarker panel and blue arrows the surplus four volatiles (numbers 7–10), with the correspondence of numbers to metabolite identities present in (b)). (**b**) Heatmap illustrating the mean levels (normalized peak areas) of metabolites included in the 10-biomarker panel. Rows correspond to the mean normalized peak area of each metabolite with the sample groups in the columns. (**c**–**f**) Partial least squares discriminant analysis (PLS-DA) scores scatter plots (UV scaling; two components) obtained for the 10-biomarker panel of (c) PCa (*n* = 18, blue squares) vs. cancer-free controls (*n* = 19, green circles), (d) PCa (*n* = 18, blue squares) vs. bladder cancer (BC) (*n* = 18, red circles), (e) PCa (*n* = 18, blue squares) vs. renal cancer (RC) (*n* = 20, yellow circles), (f) PCa (*n* = 17, blue squares) vs. cancer-free controls plus BC and RC (*n* = 58, pink circles). (**g**–**j**) Assessment of the diagnostic performance of the PLS-DA models obtained for the 10-biomarker panel of (g) PCa vs. cancer-free controls (area under the curve (AUC) = 0.95; sensitivity = 78%; specificity = 100%; accuracy = 89%), (h) PCa vs. BC (AUC = 0.88; sensitivity = 72%; specificity = 100%; accuracy = 86%), (i) PCa vs. RC (AUC = 0.89; sensitivity = 72%; specificity = 90%; accuracy = 82%), (j) PCa vs. cancer-free controls plus BC and RC (AUC = 0.90; sensitivity = 76%; specificity = 97%; accuracy = 92%).

A targeted metabolomics approach (Figure 1a) was first performed to build a partial least squares discriminant analysis (PLS-DA) model comprising only the six volatile metabolites included in the previously defined PCa biomarker panel, specifically 2,5-dimethylbenzaldehyde, 3-phenylpropionaldehyde, 4-methylhexan-3-one, dihydroedulan IA, hexanal, and methylglyoxal (MG). The classification model confirmed that this 6-biomarker panel was able to discriminate PCa from controls (Figure S1A), as validated by permutation tests (Figure S2A), with 84% sensitivity, 80% specificity, 82% accuracy, and an AUC of 0.83 (Figure S2B). However, the panel was unable to discriminate PCa from either BC (Figure S1B) or RC (Figure S1C). To overcome this limitation, an untargeted approach was conducted, seeking to identify new biomarkers able to discriminate PCa from the other urological cancers.

### 2.2. Untargeted Volatile Profiling Unveils Discriminant Metabolites among Urological Cancers

The untargeted approach consisted on the comparison of the urinary volatile profiles (VOCs and volatile carbonyl compounds (VCCs)) of PCa patients with that of BC and RC patients. Results disclosed a good separation of PCa from BC and RC in the PLS-DA models (Table S1). Overall, 50 metabolites showed a variable importance to the projection (VIP) higher than 1 comparing PCa vs. BC, from which 35 were found significantly different. Comparison of PCa vs. RC unveiled a total of 62 metabolites with VIP > 1, from which 47 were significantly different. Subsequently, only the metabolites that met the criteria of statistical significance in the three comparisons (PCa vs. BC, PCa vs. RC, and PCa vs. controls) were considered for further analysis, namely ethylbenzene, heptan-3-one, heptan-2-one, 4-(2-methylpropoxy)butan-2-one, methyl benzoate, 3-methyl-benzaldehyde, and an unknown metabolite (Figure S3; Tables S2 and S3).

### 2.3. Definition of an Improved Biomarker Panel for PCa Diagnosis

The seven candidate biomarkers disclosed by untargeted analysis were assessed for their ability to perfect an extended biomarker panel providing the best combination of sensitivity, specificity, and accuracy for PCa detection. Considering a PLS-DA based algorithm, four metabolites were selected, namely ethylbenzene, heptan-2-one, methyl benzoate, and 3-methylbenzaldehyde (Figure S4). Therefore, a final 10-biomarker panel was defined for PCa diagnosis (Figure 1b), composed by hexanal, 4-methylhexan-3-one, dihydroedulan IA and MG (significantly decreased), and 3-phenylpropionaldehyde, 2,5-dimethylbenzaldehyde, ethylbenzene, heptan-2-one, methyl benzoate, and 3-methylbenzaldehyde (significantly increased). Finally, PLS-DA models obtained with this improved biomarker panel revealed a clear separation between PCa vs. controls (Figure 1c), PCa vs. BC (Figure 1d), and PCa vs. RC (Figure 1e). Furthermore, the panel disclosed 78% sensitivity, 100% specificity, 89% accuracy, and an AUC of 0.95 for PCa vs. controls (Figure 1g); 72% sensitivity, 100% specificity, 86% accuracy, and an AUC of 0.88 for PCa vs. BC (Figure 1h); and 72% sensitivity, 90% specificity, 82% accuracy, and an AUC of 0.89 for PCa vs. RC (Figure 1i). To provide a more global perspective of the 10-biomarker panel performance, a final PLS-DA model was computed comparing PCa vs. controls plus BC plus RC (Figure 1f). This model showed a good separation between the two groups with 76% sensitivity, 97% specificity, 92% accuracy, and an AUC of 0.90 (Figure 1j). The robustness of all PLS-DA models was confirmed through permutation testing (Figure S5).

## 3. Discussion

The major novelty of this study design was the inclusion of patients with other urological cancers to evaluate the performance of the biomarker panel to discriminate not only PCa vs. cancer-free individuals, but also PCa vs. BC and RC (2nd and 3rd most common urological cancers in males, respectively) [18]. This critical point is often overlooked in traditional biomarker studies, thus disregarding the cancer site specificity of the biomarker(s). Although the performance of our 6-biomaker panel to accurately identify PCa patients vs. control subjects was confirmed, it failed to discriminate PCa from both BC and RC. This result was not completely surprising as tumor cells share some metabolic abnormalities to promote cancer cell survival and growth [11], which may make the discrimination among different cancer types difficult. Indeed, three out of the six biomarkers included in the panel (2,5-dimethylbenzaldehyde, 3-phenylpropionaldehyde, and MG) disclosed the same trend in all urological cancers vs. controls (Figure 1b), explaining the lack of discriminatory power. One well-established metabolic feature of cancer cells is increased aerobic glycolysis over oxidative respiration, which unavoidably leads to MG accumulation [19]. Particularly, MG is known as a highly toxic and reactive carbonyl compound that spontaneously glycates proteins, nucleic acids, and lipids [19]. MG detoxification is accomplished mainly by glyoxalase-1, and both high expression and activity of this enzyme have been demonstrated in PCa, BC, and RC [20], explaining the decrease in MG urinary levels in all urological cancers compared to controls.

Those results demonstrated that BC or RC patients may be false positives in a PCa screening strategy based on the 6-biomarker panel, constituting a relevant limitation in clinical practice. To overcome this, we performed an untargeted metabolomic study to look for new biomarkers capable of discriminating PCa from the other urological cancers. This approach allowed the improvement of the previously established biomarker panel through the addition of four new volatile compounds, discriminative of PCa vs. BC and RC. Thus, the improved panel included five aldehydes (hexanal, 2,5-dimethylbenzaldehyde, 3-phenylpropionaldehyde, MG, 3-methylbenzaldehyde), two ketones (4-methylhexan-3-one, heptan-2-one), two aromatic hydrocarbons (methyl benzoate and ethylbenzene), and one polycyclic organic compound (dihydroedulan IA). From these, only hexanal [21,22,23,24], MG [25], heptan-2-one [22,26], and ethylbenzene [23,27] were previously associated with cancer. Besides MG, it is difficult to ascertain the metabolic origin of these volatile compounds since they are end products of several cellular processes, including lipid peroxidation, protein carbonylation, glycation, and amino acid and lipid metabolisms [28,29,30].

In the last decade, several candidate biomarkers have been proposed using metabolomic approaches, including mainly amino acids and derivatives, organic acids, and sugars [15]. Overall, the 10-biomarker panel disclosed similar or even better performance for PCa detection compared with those candidate biomarkers. Moreover, the discrimination of PCa from other urological cancers has been understudied, comprising only one study which failed to demonstrate discriminative power [31]. Hence, the accurate classification of PCa using the 10-biomarker panel in a cohort comprising other urological cancers is an important achievement. Notably, the 10-biomarker panel outperforms not only PSA [5,6,7], but also PCA3 [9] and PHI [10] in differentiating PCa patients from controls.

These results emphasize the potential of volatile biomarkers for development of a non-invasive screening tool for clinical diagnosis. However, the analytical techniques used in metabolomic approaches might not be feasible for real-time diagnostic applications due to several limitations, such as low sample throughput, high costs, and the requirement for trained personnel and sophisticated software. Thus, future research may rely on the translation of the volatile signatures detected by GC-MS analysis to the application of a fast, cheap, and portable e-nose device. In this regard, our defined panel holds the potential for development of an optimized e-nose—with sensor arrays targeting specific PCa-related volatile compounds—for routine clinical use with high accuracy for PCa diagnosis.

## 4. Material and Methods

### 4.1. Study Population

Urine samples were collected at the Portuguese Oncology Institute of Porto from a total of 80 men, comprising 20 PCa patients, 20 BC patients, 20 RC patients, and 20 cancer-free individuals (controls) (Table S4). This study was performed in accordance with the Declaration of Helsinki and approved by the Portuguese Oncology Institute of Porto (IPO Porto) Ethics Committee (Reference 282R/2017). All subjects included in the study provided signed informed consent. Early morning voided urine samples (without fasting) were centrifuged and the supernatants were immediately frozen at −80 °C until analysis.

### 4.2. Sample Preparation for GC-MS Based Metabolomic Analysis

Preparation of urine samples and analytical conditions followed the protocol described by Lima et al. [17]. To detect a large number of volatile compounds, two different analysis procedures based on headspace solid-phase microextraction (HS-SPME) coupled to GC-MS were performed. VOCs were analyzed directly in the headspace of the urine, while VCCs (mostly aldehydes and ketones) were analyzed after a derivatization step of urine with O-(2,3,4,5,6-pentafluorobenzyl) hydroxylamine hydrochloride (PFBHA). Quality control (QC) samples (a pool of all urine samples) were analyzed at the same conditions on every eight samples, to ensure the reproducibility of the method. All samples were extracted and injected randomly.

### 4.3. GC-MS Analysis

VOCs and VCCs analyses were performed in a 436-GC (Bruker Daltonics, Billerica, MA, USA) coupled to a Bruker Scion SQ MS detector (Bruker Daltonics) and a 436-GC (Bruker Daltonics) coupled to an EVOQ TQ-MS (Bruker Daltonics), respectively. Details on software, GC and MS acquisition parameters, and metabolite identification are described in Lima et al. [17].

### 4.4. Data Pre-Processing and Statistical Analysis

Data pre-processing was performed in MZmine 2.18 [32] using the parameters previously described in Lima et al. [17]. In the untargeted models, a variable selection approach was applied to reduce the data and eliminate the variation from uncontrolled confounding factors [33]. This variable selection was based on the *t*-test (MetaboAnalyst 4.0 software) [34] and all variables with *p*-value > 0.05 were removed from the matrix. Finally, the untargeted models were scaled to pareto and the models based in the biomarker panel were scaled to unit variance (UV).

Multivariate statistical analysis (MVA) included principal component analysis (PCA) to detect trends and possible outliers, followed by partial least squares discriminant analysis (PLS-DA). In addition, the robustness of all PLS-DA models was confirmed through 7-fold cross validation and permutation test in SIMCA-P 15 (Umetrics, Umeå, Sweden).

Unpaired Student’s *t*-test with Welch correction test (normal distribution) or unpaired Mann–Whitney U-test (non-normal distribution) were applied to all metabolites with VIP higher than 1 in the untargeted models (VOCs and VCCs) (GraphPad Prism 6, San Diego, CA, USA). In addition, percentage of variation and effect size were computed for all statistically significant metabolites. The metabolites significantly altered in all comparisons (PCa vs. BC, PCa vs. RC, and PCa vs. controls) were considered specific for PCa. To select the most important metabolites among these, a PLS-DA algorithm was applied. The selected biomarker panel was used to construct a classification model and the corresponding receiver operating characteristic (ROC) curve using the MetaboAnalyst 4.0 software [34].

## 5. Conclusions

In conclusion, we propose an improved volatile urinary biomarker panel to simultaneously discriminate PCa from cancer-free subjects and carriers of other common urological cancers, holding the potential for the development of a non-invasive early detection tool for PCa that outperforms serum PSA sensitivity and accuracy. In the future, we plan to extend the PCa-specificity of this novel panel, testing larger cohorts of cancer patients and controls, including common non-urological cancers in males (e.g., lung and colorectal cancers). Furthermore, taking into consideration that benign prostate hyperplasia, urinary tract infection, and prostatitis are important confounding factors in PSA test, we also plan to include urine samples from patients with these diseases.

## Supplementary Materials

The following are available online at https://www.mdpi.com/2072-6694/12/8/2017/s1, Figure S1: PLS-DA scores scatter plots obtained for the urinary 6-biomarker panel, Figure S2: Statistical validation and assessment of the diagnostic performance of the PLS-DA model obtained for the 6-biomarker panel, Figure S3: Boxplots from all metabolites simultaneously significantly different, Figure S4: VIP scores computed through a PLS-DA based algorithm to select the metabolites that best discriminate the groups, Figure S5: Statistical validation of the PLS-DA model obtained for the 10-biomarker panel, Table S1: Seven-fold cross validation parameters obtained for PLS-DA models, Table S2: Univariate statistical analysis results of VOCs and VCCs significantly altered in PCa group compared to BC, RC, and cancer-free controls., Table S3: Characterization of the VOCs and VCCs significantly altered in PCa group compared to BC, RC, and cancer-free controls, Table S4: Demographic and clinical data of PCa, BC, and RC patients and cancer-free controls.
