# Supplementary material for: A Panel of Urinary Volatile Biomarkers for Differential Diagnosis of Prostate Cancer from Other Urological Cancers
Source: Cancers (Basel). 2020 Jul 23;12(8):2017. doi: 10.3390/cancers12082017 (PMC7464354; doi:10.3390/cancers12082017)
Supplement: Supplementary file 1 [file cancers-12-02017-s001.pdf]

SUPPLEMENTARY FIGURES

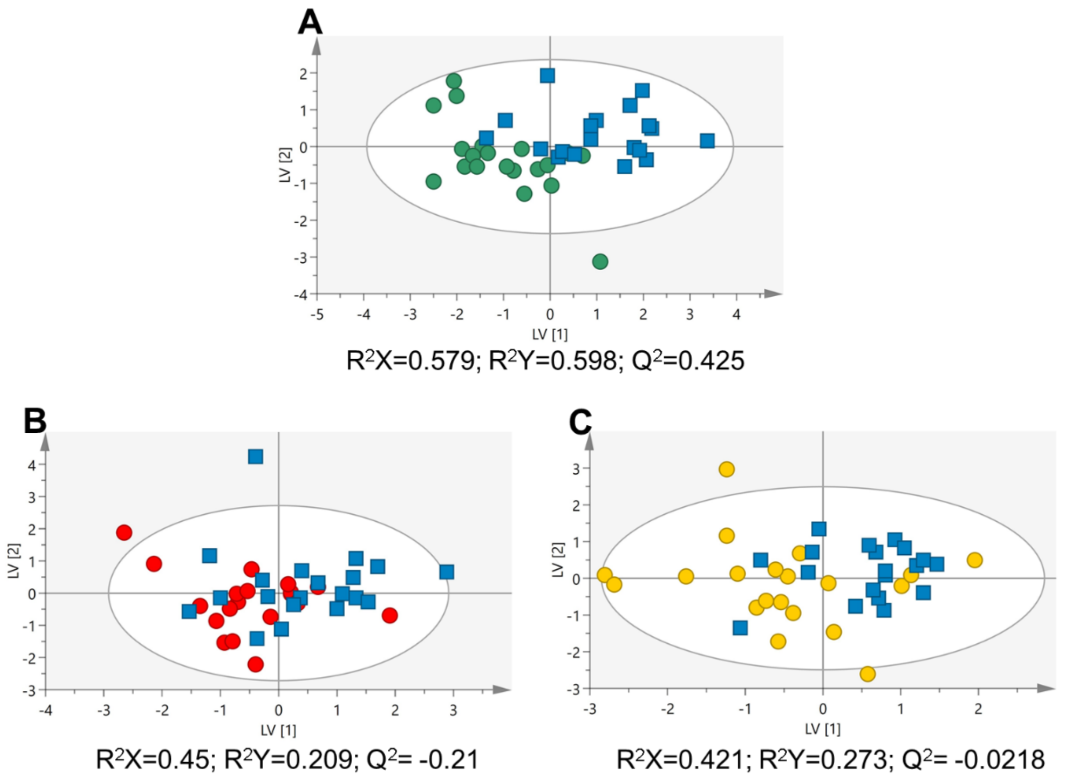

Supplementary Figure 1. PLS-DA scores scatter plots (UV scaling; 2 components) obtained for the urinary 6-biomarker panel of **(A)** PCa patients (n = 19, blue squares) vs. cancer-free controls (n = 20, green circles); **(B)** PCa (n = 20, blue squares) vs. BC (n = 19, red circles); and **(C)** PCa (n = 19, blue squares) vs. RC (n = 20, yellow circles).

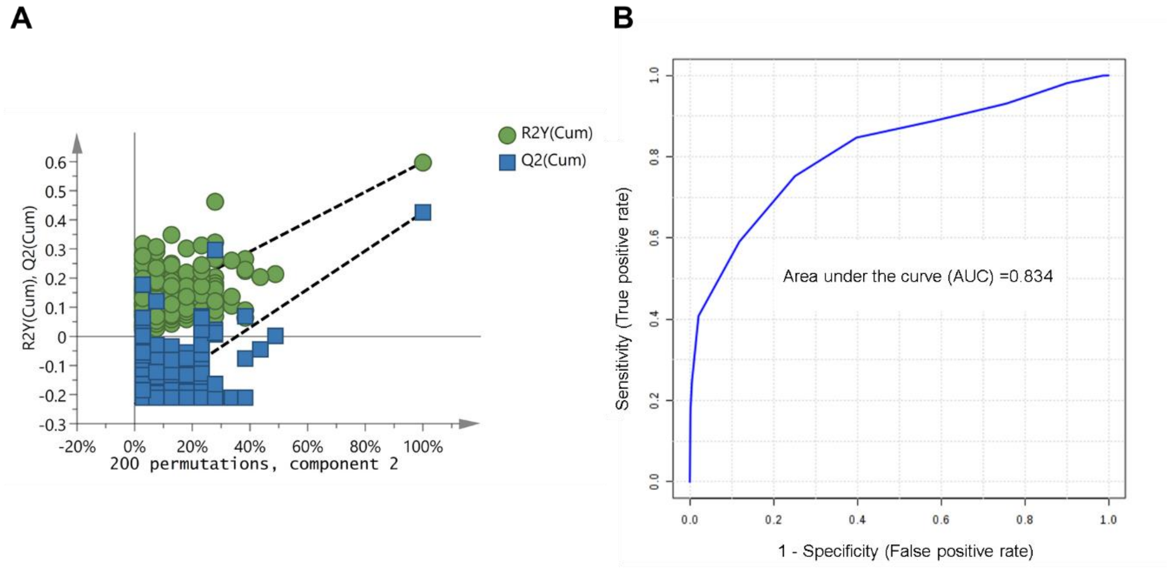

Supplementary Figure 2. (A) Statistical validation of the PLS-DA model obtained for the 6-biomarker panel, by permutation testing (200 permutations; 2 components) PCa vs. cancer-free controls [Intercepts: R^2 = (0.0, 0.0866), Q^2 = (0.0, -0.234)]; **(B)** Assessment of the diagnostic performance of the PLS-DA model obtained for the 6-biomarker panel through receiver operating characteristic (ROC) curve, PCa vs. controls (AUC = 0.834; sensitivity = 84%; specificity = 80%; accuracy = 82%).

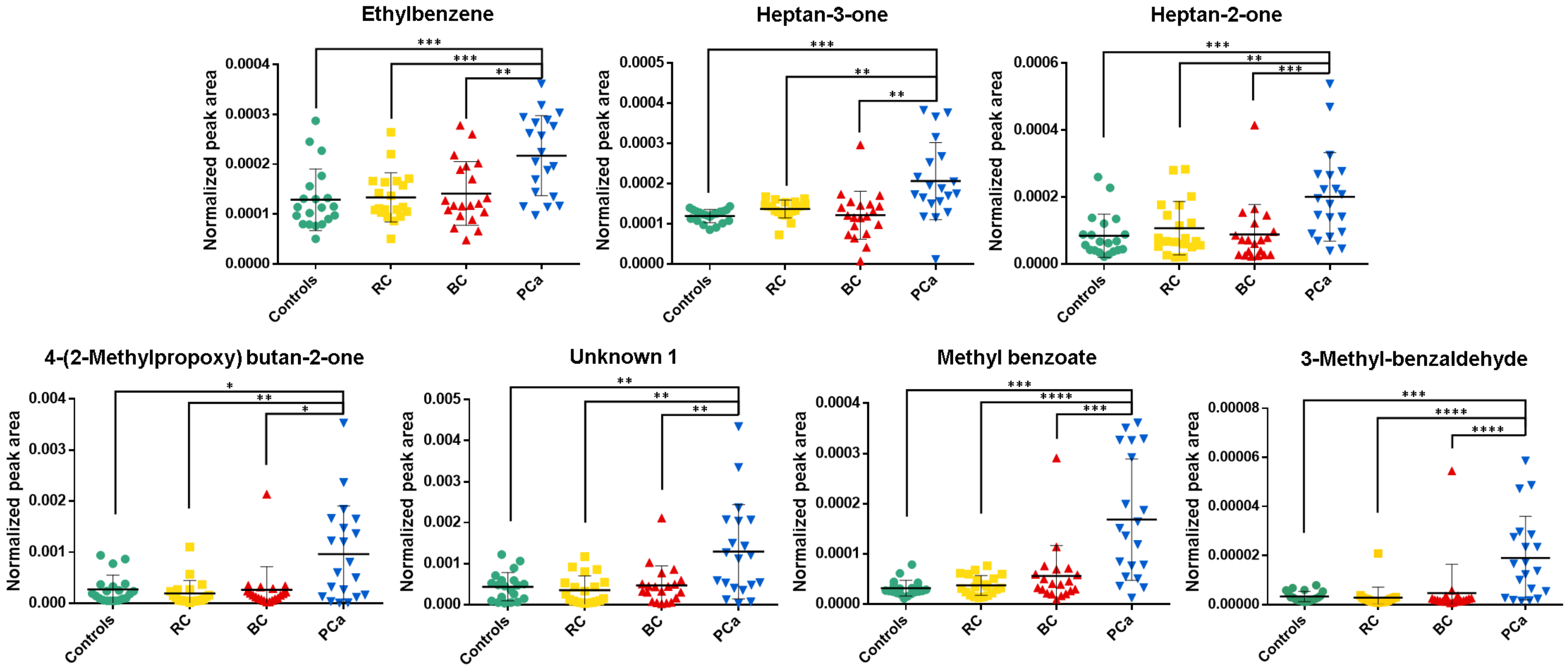

Supplementary Figure 3. Boxplots from all metabolites that were simultaneously significantly different between PCa vs. BC, PCa vs. RC and PCa vs. cancer-free controls (****p-value < 0.0001, ***p-value < 0.001, **p-value < 0.01, *p-value < 0.05).

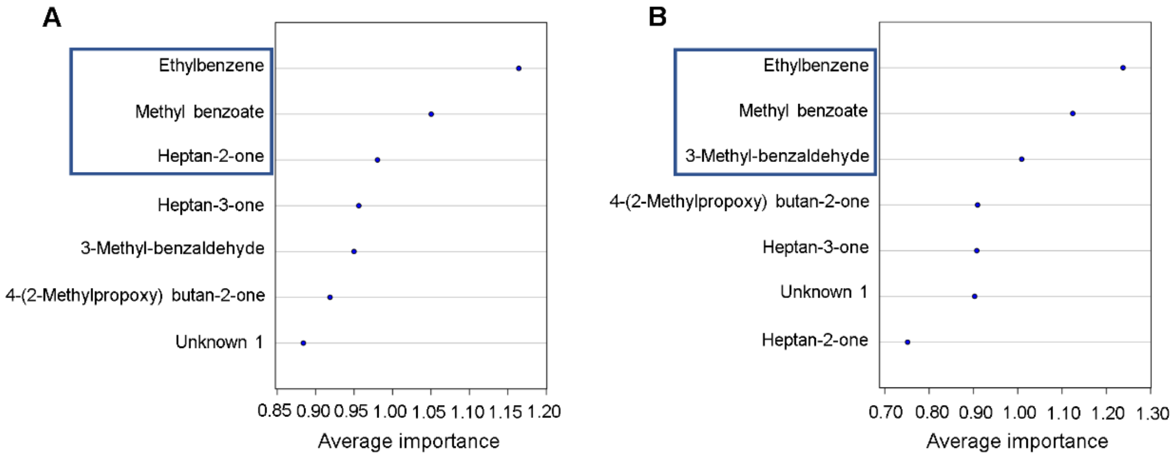

Supplementary Figure 4. VIP scores computed through a PLS-DA based algorithm to select the metabolites that best discriminate the groups: **(A)** PCa vs. BC; **(B)** PCa vs. RC.

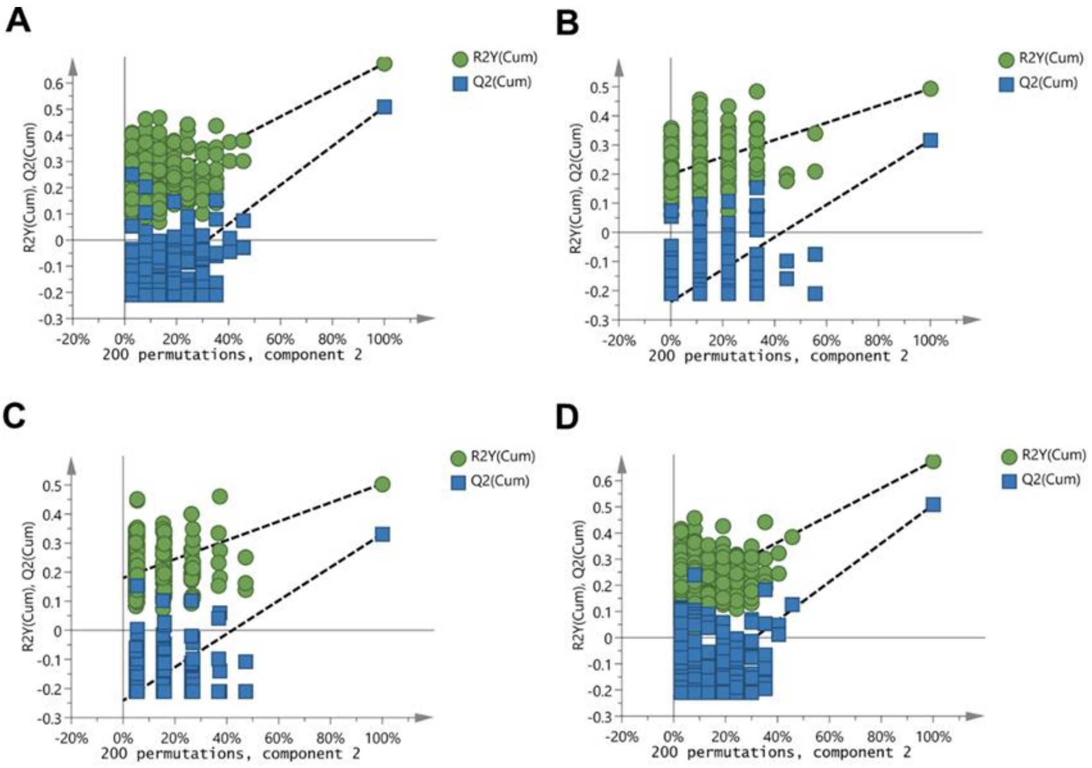

Supplementary Figure 5. Statistical validation of the PLS-DA model obtained for the 10-biomarker panel, by permutation testing (200 permutations; 2 components). **(A)** PCa vs. controls [Intercepts: R^2 = (0.0, 0.167), Q^2 = (0.0, -0.237)]; **(B)** PCa vs. BC [Intercepts: R^2 = (0.0, 0.2), Q^2 = (0.0, -0.238)]; **(C)** PCa vs. RC [Intercepts: R^2 = (0.0, 0.18), Q^2 = (0.0, -0.241)]; **(D)** PCa vs. controls plus BC and RC [Intercepts: R^2 = (0.0, 0.157), Q^2 = (0.0, -0.229)].

SUPPLEMENTARY TABLES

Supplementary Table 1. 7-Fold cross validation parameters obtained for PLS-DA models of VOCs and VCCs in the untargeted approach.

	VOCs				VCCs			
**Comparison**	**LV**	**R^2X**	**R^2Y**	**Q^2**	**LV**	**R^2X**	**R^2Y**	**Q^2**
**PCa *vs.* BC**	2	0.544	0.773	0.655	2	0.414	0.742	0.554
**PCa *vs.* RC**	2	0.403	0.772	0.477	2	0.702	0.628	0.394

Supplementary Table 2. Univariate statistical analysis results of VOCs and VCCs significantly altered in PCa group compared to BC, RC and cancer-free controls.

		**PCa *vs.* BC**			**PCa *vs.* RC**			**PCa *vs.* Controls**		
Chemical name (IUPAC)	***Protocol***	***p-value***	***Variation ± uncertainty (%)***	***Effect size ± ES_{SE}***	***p-value***	***Variation ± uncertainty (%)***	***Effect size ± ES_{SE}***	***p-value***	***Variation ± uncertainty (%)***	***Effect size ± ES_{SE}***
Ethylbenzene	VOCs	0.0021	91.15 ± 16.80	0.83 ± 0.45	0.0004	62.77 ± 12.00	1.23 ± 0.67	0.0002	68.59 ± 13.07	1.21 ± 0.66
Heptan-3-one	VOCs	0.0021	69.75 ± 15.41	1.04 ± 0.65	0.0048	50.64 ± 12.83	0.98 ± 0.64	0.0007	72.56 ± 13.35	1.24 ±0.67
Heptan-2-one (2-Heptanone)	VOCs	0.0005	126.37 ± 24.58	0.98 ± 0.64	0.0082	87.09 ± 22.36	0.84 ± 0.63	0.0003	137.2 ± 23.00	1.10 ±0.65
4-(2-Methylpropoxy) butan-2-one	VOCs	0.0124	264.40 ± 37.95	0.93 ± 0.64	0.0035	398.07 ± 37.60	1.10 ± 0.65	0.0210	251.4 ± 35.36	0.98 ±0.64
Methyl benzoate	VOCs	0.0002	200.05 ± 26.93	1.15 ± 0.66	<0.0001	350.68 ± 26.59	1.48 ± 0.69	<0.0001	430.1 ± 27.21	1.56 ± 0.70
Unknown 1	VOCs	0.0061	175.99 ± 31.57	0.92 ± 0.64	0.0013	267.36 ± 32.60	1.09 ± 0.65	0.0075	195.7 ± 30.10	0.99 ± 0.65
3-Methyl-benzaldehyde	VCCs	<0.0001	305.49 ± 39.22	0.96 ± 0.64	<0.0001	572.98 ± 36.09	1.27 ± 0.67	0.0003	476.8 ± 34.50	1.27 ± 0.67

The statistical significance (p-values), percentage of variation, effect size (ES), standard error (ES_{SE}) are represented for each volatile compound. .

Supplementary Table 3. Characterization of VOCs and VCCs significantly altered in PCa group compared to BC, RC and cancer-free controls. They are characterized by their IUPAC name, retention time, characteristic ions (*m/z*), Kovat indices (KI) from literature, experimental KI, NIST R-match, CAS registry number and human metabolome database (HMDB) code.

**Chemical name (IUPAC)**	**Protocol**	**Retention time**	***m/z***	**KI from literature**	**Experimental KI**	**R-match**	**CAS number**	**Identification Level**	**HMDB**
Ethylbenzene	VOCs	6.44	91; 106; 51; 65; 77;78; 92; 50; 105	855	-	853	100-41-4	L1	HMDB0059905
Heptan-3-one	VOCs	7.10	57; 85; 72; 114	877	884	845	106-35-4	L2	HMDB0031482
Heptan-2-one	VOCs	7.20	58; 71; 59	891	887	835	110-43-0	L1	HMDB0003671
4-(2-Methylpropoxy) butan-2-one	VOCs	8.47	71;72; 57; 55; 101; 89	964	-	735	31576-33-7	L2	-
Methyl benzoate	VOCs	13.29	105; 77; 55; 51; 136; 57; 71; 50	1094	-	856	93-58-3	L1	HMDB0033968
Unknown 1	VOCs	10.75	57; 59; 69; 89; 56; 71; 87; 58	-	1009	-	-	L4	-
3-Methyl-benzaldehyde	VCCs	29.98	315; 77; 91; 182; 65; 79; 285; 78, 89	1845	-	788	620-23-5	L1	HMDB0029637

L1: Identified metabolites (GC-MS analysis of the metabolite of interest and a chemical reference standard of suspected structural equivalence, with all analyses performed under identical analytical conditions within the same laboratory); L2: Putatively annotated compounds (spectral (MS) similarity with NIST database); L4: Unidentified.

Supplementary Table 4. Demographic and clinical data of prostate cancer (PCa), bladder cancer (BC) and renal cancer (RC) male patients and cancer-free male controls included in this study.

Characteristics	PCa	BC	RC	Controls
Number of subjects	20	20	20	20
Mean Age ± SD (years)	67 ± 8.1	69 ± 8.6	71 ± 7.7	58 ± 2.8
PSA (ng/mL), *n* (%)				
<4	1 (5%)	-	-	-
4-10	7 (35%)	-	-	-
>10	4 (20%)	-	-	-
Not available	8 (40%)	20 (100%)	20 (100%)	20 (100%)
Gleason score, *n* (%)				
≤6	7 (35%)	-	-	-
=7	9 (45%)	-	-	-
≥10	3 (15%)	-	-	-
Not available	1 (5%)	20 (100%)	20 (100%)	20 (100%)
Clinical stage, *n* (%)				
0	-	9 (47%)	2 (10%)	-
I	7 (35%)	6 (32%)	11 (55%)	-
II	3 (15%)	2 (11%)	1 (5%)	-
III	2 (10%)	-	5 (25%)	-
IV	6 (30%)	2 (11%)	1 (5%)	-
Not available	2 (10%)	-	-	-
